# Downregulation of miR-146a Contributes to Cardiac Dysfunction Induced by the Tyrosine Kinase Inhibitor Sunitinib

**DOI:** 10.3389/fphar.2019.00914

**Published:** 2019-08-23

**Authors:** Li Shen, Congxin Li, Hua Zhang, Suhua Qiu, Tian Fu, Yanfang Xu

**Affiliations:** Department of Pharmacology, Hebei Medical University, The Key Laboratory of New Drug Pharmacology and Toxicology, Hebei Province, The Key Laboratory of Neural and Vascular Biology, Ministry of Education, Shijiazhuang, China

**Keywords:** sunitinib, contractile dysfunction, miR-146a, PLN, ANK2

## Abstract

The main adverse effect of tyrosine kinase inhibitors, such as sunitinib, is cardiac contractile dysfunction; however, the molecular mechanisms of this effect remain largely obscure. MicroRNAs (miRNAs) are key regulatory factors in both cardiovascular diseases and the tyrosine kinase pathway. Therefore, we analyzed the differential expression of miRNAs in the myocardium in mice after exposure to sunitinib using miRNA microarray. A significant downregulation of miR-146a was observed in the myocardium of sunitinib-treated mice, along with a 20% decrease in left ventricle ejection fraction (LVEF). The downregulation of miR-146a was further validated by RT-qPCR. Among the potential targets of miR-146a, we focused on *Pln* and *Ank2*, which are closely related to cardiac contractile dysfunction. Results of luciferase reporter assay confirmed that miR-146a directly targeted the 3′ untranslated region of *Pln* and *Ank2*. Significant upregulation of PLN and ANK2 at the mRNA and protein levels was observed in the myocardium of sunitinib-treated mice. Cardiac-specific overexpression of miR-146a prevented the deteriorate effect of SNT on calcium transients, thereby alleviating the decreased contractility of human induced pluripotent stem cell-derived cardiomyocytes (hiPSC-CMs). SiRNA knockdown of PLN or ANK2 prevented sunitinib-induced suppression of contractility in hiPSC-CMs. Therefore, our *in vivo* and *in vitro* results showed that sunitinib downregulated miR-146a, which contributes to cardiac contractile dysfunction by regulating the downstream targets PLN and ANK2, and that upregulation of miR-146a alleviated the inhibitory effect of SNT on cardiac contractility. Thus, miR-146a could be a useful protective agent against sunitinib-induced cardiac dysfunction.

## Introduction

Small molecule inhibitors targeting receptor tyrosine kinases have markedly improved the life expectancy of cancer patients (Motzer et al., 2006). However, many of these agents have unintended consequences on the cardiovascular system, including hypertension, left ventricular (LV) dysfunction, and chronic heart failure (Richards et al., 2011). Sunitinib (SNT) is a multi-targeted oral tyrosine kinase inhibitor widely used to treat solid tumors. Clinical studies have provided evidence that approximately 20% of patients receiving SNT suffer from reduced LV ejection fraction (LVEF), and 8–15% of patients develop congestive heart failure (CHF) (Chu et al., 2007). To date, a specific protective drug or approach to prevent the side effects of SNT is still not available because information on the molecular mechanisms of cardiac contractile dysfunction remains limited (Faivre et al., 2007; Chen et al., 2008). Therefore, identifying the molecular mechanisms that regulate this injury will help develop advanced interference treatments. Recent studies have shown that decreased intracellular Ca^2+^ transition contributes to SNT-induced negative inotropic effect (Rainer et al., 2012), which may result from its effect on phospholamban (PLN), a key regulator of sarcoplasmic endoreticulum Ca^2+^-ATPase (SERCA) (Schneider et al., 2018). However, the detailed molecular mechanism underlying the functional effect of SNT on PLN is unknown.

Since Lin-4 was first found (Lee et al., 1993), microRNAs (miRNAs) have been confirmed as a powerful tool in examining post-transcriptional gene expression. MiRNAs are small non-coding RNAs that play an important role in pathophysiological processes. In the cytoplasm, miRNAs inhibit transcription or translational repression by targeting the 3′ untranslated region (3′UTR) of mRNAs (Pasquinelli, 2012). Owing to their important role as regulators of gene expression, abnormal expression of miRNAs has been assessed in various diseases, and thus may act as therapeutic targets (Kalozoumi et al., 2014). Numerous studies have confirmed that miRNAs play a key role in cardiovascular diseases (Creemers et al., 2012; Gomes da Silva and Silbiger, 2014); thus, overexpression and downregulation of miRNAs may serve as promising therapeutic approaches for patients with heart diseases (Ikeda et al., 2007). Many cardiotoxic drugs affect the expression level of miRNAs in the heart (Skala et al., 2019), and differential expression of miRNAs is involved in the pathogenesis of heart failure (Wong et al., 2016). MiRNAs may also influence calcium cycling; in addition, Ca^2+^-related miRNAs may serve as therapeutic targets in the treatment of heart failure. (Choi et al., 2014).

Considering the important role of miRNAs in gene expression, we hypothesized that miRNAs may contribute to SNT-induced cardiac contractile dysfunction by targeting intracellular Ca^2+^ cycling proteins, including PLN. Therefore, in this study, we analyzed alterations in the expression of miRNAs in mouse myocardium after exposure to SNT. Next, we focused on miR-146a, which was the most downregulated miRNA following SNT treatment, and assessed the protective effect of miR-146a upregulation on SNT-induced cardiac contractile dysfunction *in vivo* and *in vitro*.

## Materials and Methods

### Animals

Male C57 mice (20–25 g) were purchased from Vital River Laboratories (Beijing, China), and were housed under pathogen-free conditions and fed a standard mouse chow with free access to water and food. The experiments were performed in the Department of Pharmacology, Hebei Medical University. All animal care and experimental procedures were approved by the Animal Care and Ethical Committee of Hebei Medical University (Shijiazhuang, China). In the *in vivo* study, the mice received a daily oral administration of SNT 40 mg/kg for 1 week.

### Echocardiography

LV contractile function was assessed using a high-resolution ultrasound imaging system (Vevo2100 imaging system; FUJIFIUM VisualSonics Inc., Toronto, Canada). Mice were anesthetized with 1–2% isoflurane and then underwent transthoracic two dimensional (2D) guided M-mode echocardiography with an 18–38MHz probe. Left ventricular ejection fraction (LVEF) and fractional shortening (FS) were measured.

### Cell Culture and Monitoring by CardioExcyte 96

Human induced pluripotent stem cell-derived cardiomyocytes (hiPSC-CMs) were purchased from Beijing Cellapy Biotechnology Co., Ltd. (Beijing, China). Cells were stored in liquid nitrogen until thawed, and cultured according to the manufacturer’s instructions. CardioExcyte 96 sensor plates (NSP-96) were incubated in 10 μg/ml fibronectin (Sigma Aldrich, Steinheimn, Germany) for 2 h at 37°C. The cells were then seeded on NSP-96 plates (3×10^4^ cells/well). The complete medium was replaced after 48 h, and half of the medium’s volume was replaced each day.

CardioExcyte 96 (Nanion Technologies, Germany) has been used to study the pharmacological effects of drugs on contractility through impedance measurements (Doerr et al., 2015). In this study, impedance amplitude was measured to assess the contractility of hiPSC-CMs, following a previous study (Bot et al., 2018). The stable impedance recordings from monolayer cells in each well were obtained for 5–7 days, and the effect of the drugs were then examined. Subsequently, the mean beat was automatically obtained by fitting a sequence of beats into a single beat, yielding a more descriptive and condensed view. On the day of drug administration, the cell culture media was completely removed from the wells and replaced with a volume of drug-containing fresh media. The cells were allowed to re-equilibrate for 2–3 h, and online parameters were monitored to ensure a stable baseline.

### Quantitative mRNA and miRNA Measurements

Total RNA was isolated using TRIzol reagent (Invitrogen, Carlsbad, CA, USA), reverse transcribed using a reverse transcription kit (TaKaRa, PrimeScript™ RT reagent Kit), and normalized with GAPDH. A MiRNA Kit (Omega Bio-tex, R6842-01) was used to extract miRNA. Maxima SYBRGreen/ROX qPCR Master Mix (ZF102-2; ZOMANBIO, Beijing, China) was used for reverse transcription-quantitative PCR (RT-qPCR) to determine the relative quantification of miRNAs. MiRNAs were normalized with U6 snRNA (7300HT Fast Real-Time PCR system; Applied Biosystems, USA). The following primers were used: *Pln* forward 5′-CCAGTGAGCTTTCCTGCGTA-3′, reverse 5′-AGTTTGCAGGTCTGGAGTGG-3′; and *Ank2* forward 5′-CAACTTTCTCGCCATGTCTGC-3′, reverse 5′-CCAAGAGTGACTGGGGTTTGA-3′. Each sample was analyzed in triplicates, and the analysis was performed using the 2^-ΔΔ^Ct method.

### miRNA Microarray

Analysis of miRNA expression was performed on the total RNA extracted from left ventricle tissue samples of mice administered vehicle or SNT using Trizol reagent or Trizol LS reagent (Invitrogen), respectively. Microarray assay and data analysis were performed at Shanghai Biotechnology Corporation (Shanghai, China). Briefly, 200 ng of total RNA extracted from the myocardium was fluorescent-labeled with Cyanine3-pCp using miRNA Complete Labeling and Hyb Kit (Agilent Technologies, Santa Clara, CA, USA). The labeled samples were then concentrated and hybridized using Hybridization Chamber gasket slides (Agilent Technologies). Arrays were scanned using an Agilent chip scanner (G2565CA), and image analysis was performed using the Agilent Feature Extraction (v10.7) software (Agilent Technologies), followed by data normalization using the Agilent GeneSpring software (Agilent Technologies). Five miRBase databases were used to predict the target genes of differentially expressed miRNAs, including the microRNA (http://www.microrna.org/microrna/home.do), TargetMiner (http://www.isical.ac.in/∼bioinfo_miu/targetminer20.htm), TarBase (http://diana.imis.athena-innovation.gr/DianaTools/index.php?r=tarbase/index), miRDB (http://mirdb.org/miRDB/index.html), and RNA22 (https://cm.jefferson.edu/rna22/) databases.

### Luciferase Assays

Recombinant luciferase reporter plasmids containing sequences of potential miR-146a-5p binding sites in the 3′UTR of the *Pln* or *Ank2* genes were constructed. Using a site-directed mutagenesis kit (TransGen, Beijing, China), miR-146a-5p and the *Pln* complementary binding sequence, UUCAGUUC, were replaced with TTUCAAUC, whereas miR-146a-5p and the *Ank2* complementary binding sequence, CAGUUCUC, were replaced with UTUACATU to construct mutant recombinant luciferase reporter plasmids. Human embryonic kidney (HEK) 293 cells were cultured in 24-well plates. After 24 h, the cells (estimated density, 70%) were co-transfected with 100 ng of recombinant luciferase reporter plasmids (wild-type or mutants) for *Pln* or *Ank2* using riboFECT CP Transfection Kit. Both the miRNA mimic and plasmid vectors were purchased from Guangzhou RiboBio Co., Ltd. After 48 h of co-transfection, the cells were lysed with passive lysis buffer and subjected to luciferase activity assay using FLUOstar Omega Microplate Reader in a GLOMAX 96 microplate luminometer (E1910; Promega, USA).

### Immunoblotting Assay

Left ventricular tissue samples of C57 mice were extracted using the Total Protein Extraction Kit (SD-001; Invent Biotechnologies, USA). Protease inhibitor and phosphatase inhibitors were added 1:100 to SD-001 lysis buffer. A BCA assay (Beyotime Biotechnology, China) was used to estimate protein concentration. After denaturing, 20 μg protein was fractionated on 15% SDS-PAGE to identify PLN and p-PLN, and 30 μg protein was loaded on 8% SDS-PAGE to identify Ankyrin2 (ANK2), followed by transferal to PVDF membranes (Millipore, 0.22 μm) for 2 h (PLN and p-PLN) or 3 h (ANK2) at 4°C. Membranes were blocked with 5% BSA in TBST for 4 h, followed by incubation with the primary antibodies including anti-PLN (GeneTex, GTX109254, USA, diluted to 1:2,000), anti-p-PLN (Affinity, AF7278, China, diluted to 1:500), ANK2 (Arigobio, S105-13, China diluted to 1:1,000) anti-ryanodine receptor 2 (RYR2) (Abcam, ab2827, USA, diluted to 1:500) and anti-SERCA (Abcam, ab2861, USA, diluted to 1:500) at 4°C 12h. After three rounds of washing (15 min) with TBST, incubation with secondary antibodies (goat anti-rabbit,1:5,000; goat anti-mouse, 1:5,000, Rockland Immunochemicals, USA) was performed for 1 h at 37°C. The signals were identified using an Odyssey Infrared Imaging System (LICOR 9120; Li-COR, Lincoln, NE, USA). Tubulin (Antibody Revolution, ARH4207, diluted to 1:500) was used to normalize the protein bands in each sample.

### RNA Interference for PLN and ANK2

A small interfering RNA (siRNA) duplex against PLN or ANK2 was designed and constructed by Ribobio (Guangdong, China). Next, hiPSC-CMs were transfected with siRNA at a final concentration of 100 nM using Lipofectamine RNAiMax (Invitrogen) according to the manufacturer’s instruction. A non-matching siRNA was used as a negative control. After siRNA transfection for 48 h, the cells were then incubated with SNT (3 µM) for 24 h. Cellular contractility was then determined by CardioExcyte 96 apparatus.

### Calcium Transient Measurements

hiPSC-CMs were seeded in fibronectin-coated (30 μg/ml) glasses (6 mm, 1 × 10^4^ cells/glass) and cultured for 5–7 days. The cells were loaded with 5 µM of the fluorescent Ca^2+^ indicator Rhod-2 AM and 0.02% Pluronic F-127 in Tyrode’s solution for 20 min at 37°C, and then washed with Tyrode’s solution (37°C). Spontaneous Ca^2+^ transients were obtained under confocal microscopy using a single-cell line scan model (TCS-SP5; Leica Microsystems, Germany). Rhod-2 AM was excited using a 552-nM laser light, and the emitted fluorescence was recorded at 581 nM.

### Enzyme-Linked Immunosorbent Assay (ELISA)

An ELISA kit (E03C0124; Blue Gene, Shanghai China) was used to measure serum levels of cTNT in mice.

### Construction of miR-146a Into Virus Vectors

miR-146a was constructed into two expression vectors, namely recombinant adeno-associated virus serotype 9 (AAV9) and adeno virus (AV), by Vigene Biosciences (ShanDong, China). The mice were injected with AAV9 (either miR-146a or control vector) (1×10^11^ viral particles) *via* the tail vein. Five weeks after gene delivery, the mice were treated with SNT (40 mg/kg/day). The regular beating of cultured hiPSC-CMs were observed at approximately 7 days after recovery. Next, hiPSC-CMs [multiplicity of infection (MOI) value, 10] were infected with AV to overexpress miR-146a. We replaced the medium with fresh medium at 2 h after the addition of AV.

### Drugs and Compounds

SUTENT^®^ (sunitinib malate; Pfizer, USA) for *in vivo* experiments was stored below 25°C, and suspended in carboxy methyl cellulose sodium before use. For *in vitro* experiments, stock solutions of sunitinib malate (MedChem Express, Princeton, USA) were prepared in autoclaved ultra-pure water and stored at -80°C. The stock solutions were diluted to a final concentration of 3 μM in culture medium immediately before use. According to a previous research (Doherty et al., 2015) and our pilot experiment, SNT at 3 μM evoked evident inhibitory effect on contraction of hiPSC-CMs; thus, we used this concentration for our current *in vitro* experiments.

### Statistics

All data were presented as mean ± S.E.M. Group comparisons were performed with unpaired Student’s *t*-tests (for single two-group comparisons) or ANOVA with Dunnett’s *post-hoc* test (for multiple-group comparisons). Statistical analysis was carried out using the SPSS statistical software version 20 (SPSS, Inc). A *P* value of < 0.05 was considered significant.

## Results

### MiR-146a is Downregulated in the Myocardium of Mice Treated With SNT

**Figure 1:**
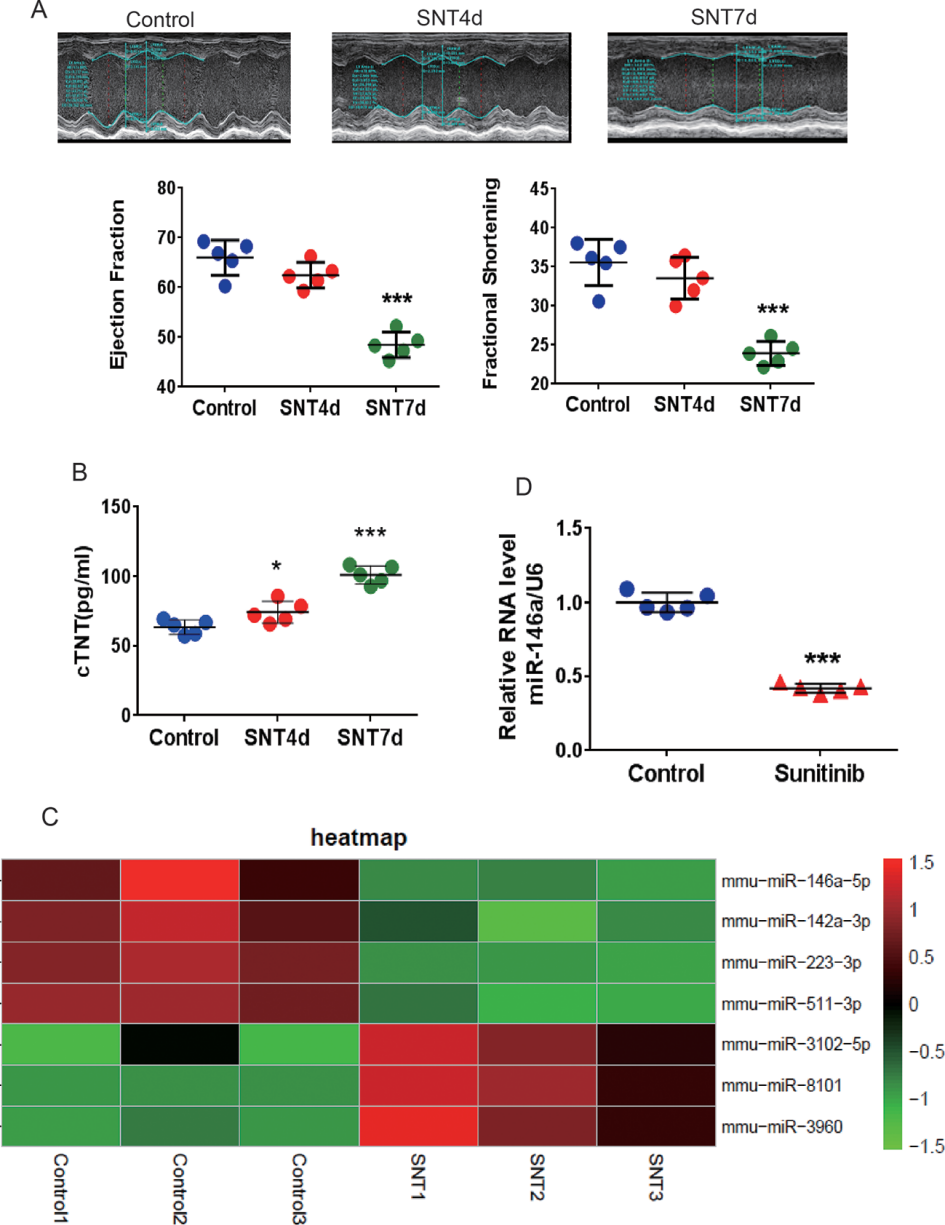
SNT induced cardiac contractile dysfunction and abnormal expression of miRNA. **(A)** For the C57mouse administered sunitinib (40 mg/kg/d), E-echocardiography showed a significant decrease in cardiac contractile function on day 7. **(B)** The level of the serum marker cTNT was increased after sunitinib-treatment. **(C)** Summary heat map of differences in miRNA expression in the left ventricle tissue of mice 7 days after sunitinib administration. **(D)** RT-qPCR results showing that sunitinib induced the downregulation of miR-146a. Values are normalized to the level of U6. *P < 0.05, ***P < 0.001 *vs* Control.

As shown in **Figure 1A**, M-mode echocardiography revealed a trend of decreasing EF and FS on day 4 after SNT treatment. Significant decreases in EF and FS were observed on day 7, along with a marked increase in serum cTNT levels (**Figure 1A, B**). Evidently, SNT treatment for 7 days evoked cardiac contractile dysfunction in mice. Mouse left ventricle tissues were then subjected to microarray assaying, and the results revealed SNT-induced miRNA alterations. The complete heatmap of differentially expressed miRNAs is shown in **Figure S1**. The data can be found in the GEO repository (GSE125952) (https://www.ncbi.nlm.nih.gov/geo/query/acc.cgi?acc).

Heat map analysis showed over two-fold dysregulation of miR-146a, miR-223, miR-511, miR-3102, miR-8101, miR-960, and miR-142a in SNT-treated mice (**Figure 1C**). MiR-146a, a human homologous gene, showed the most significant differential expression. Previous study has shown that abnormal expression of miR-146a is involved in cardiac contractile dysfunction and abnormal calcium cycling (Watson et al., 2015). The results of RT-qPCR further confirmed that miR-146a was significantly downregulated in the myocardium of mice treated with SNT for 7 days (**Figure 1D**). Thus, we focused on miR146a in the subsequent studies.

### Sunitinib Enhances *Pln* and *Ank2* Expression by Downregulating miR-146a

**Figure 2:**
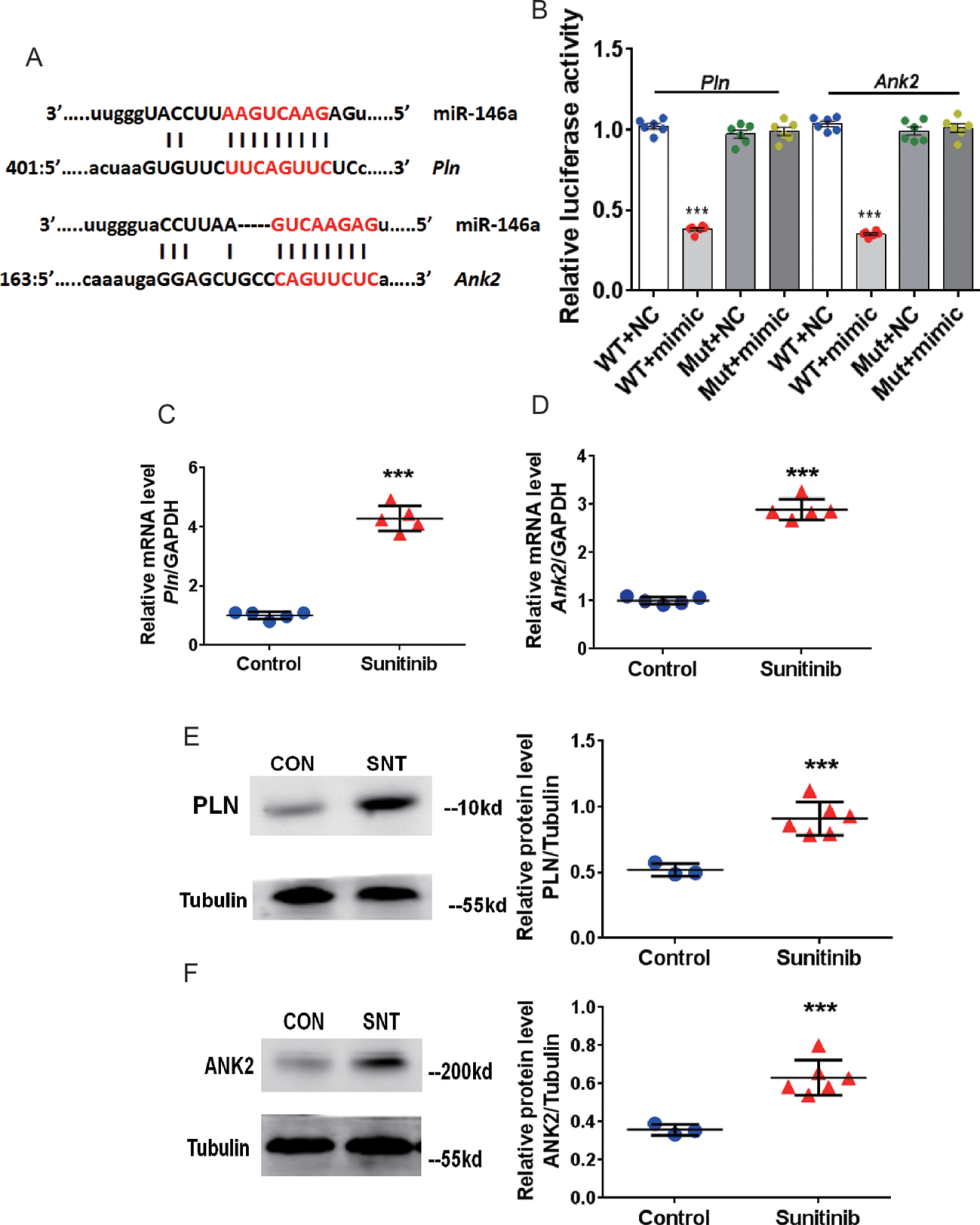
SNT enhanced PLN and ANK2 expression by downregulating miR-146a. **(A)** Representation of miR-146a and mRNA binding site for the two targets predicted by TargetScan. **(B)** Relative luciferase activity measured in HEK293 cell **(C**–**D)** *Pln* and *Ank2* mRNA levels after sunitinib treatment. RT-qPCR results were normalized to GAPDH. **(E**–**F)** Representative Western Blot bands for PLN and ANK2 protein expression and corresponding summary data after sunitinib treatment. Protein bands were normalized to Tubulin. ***P < 0.001 *vs* Control.

We then identified the targets of miR-146a. Analysis using the TargetScan software (www.targetscan.org) revealed two potential target genes, *Pln* and *Ank2*. The matching positions for miR-146a within the 3′UTR of *Pln* and *Ank2* are shown in **Figure 2A**. The *Pln* gene encodes phospholamban (PLN), which regulates cardiac SERCA *via* reversible phosphorylation. Cardiac-specific overexpression of PLN can alter calcium kinetics and influence cardiomyocyte mechanics (Kranias and Hajjar, 2012). Inhibition of PLN prevents progressive cardiac dysfunction and pathological remodeling (Iwanaga et al., 2004). The *Ank2* gene encodes ankyrin-B (ANK2), which modulates the sarcolemmal L-type Ca^2+^ current and inositol triphosphate (IP3) (Mohler et al., 2005). Some researchers found that the mRNA level of ANK-2 increases after heart damage (Hund et al., 2009). Therefore, we performed a luciferase assay using the HEK293 cell line to validate the target genes, *Pln* and *Ank2*, using special recombinant luciferase reporter plasmids containing the respective 3′UTR downstream targets of the luciferase reporter gene. Following co-transfection with the corresponding 3′UTR vector and miR-146a mimic, an evident reduction in luciferase activity was observed for both *Pln* and *Ank2*. In contrast, luciferase activity was unchanged following co-transfection with the mutant 3′UTR vectors (**Figure 2B**). Furthermore, a significant upregulation of PLN and ANK2 at the mRNA (**Figure 2C, D**) and protein (**Figure 2E, F**) levels was observed in the myocardium of mice treated with SNT for 7 days. Taken together, the results showed that SNT evoked significant downregulation of miR-146a by specifically targeting *Pln* and *Ank2*.

### Overexpression of miR-146a *In Vivo* Attenuates SNT-Induced Cardiac Dysfunction by Decreasing Abnormal Expression of PLN and ANK2

**Figure 3:**
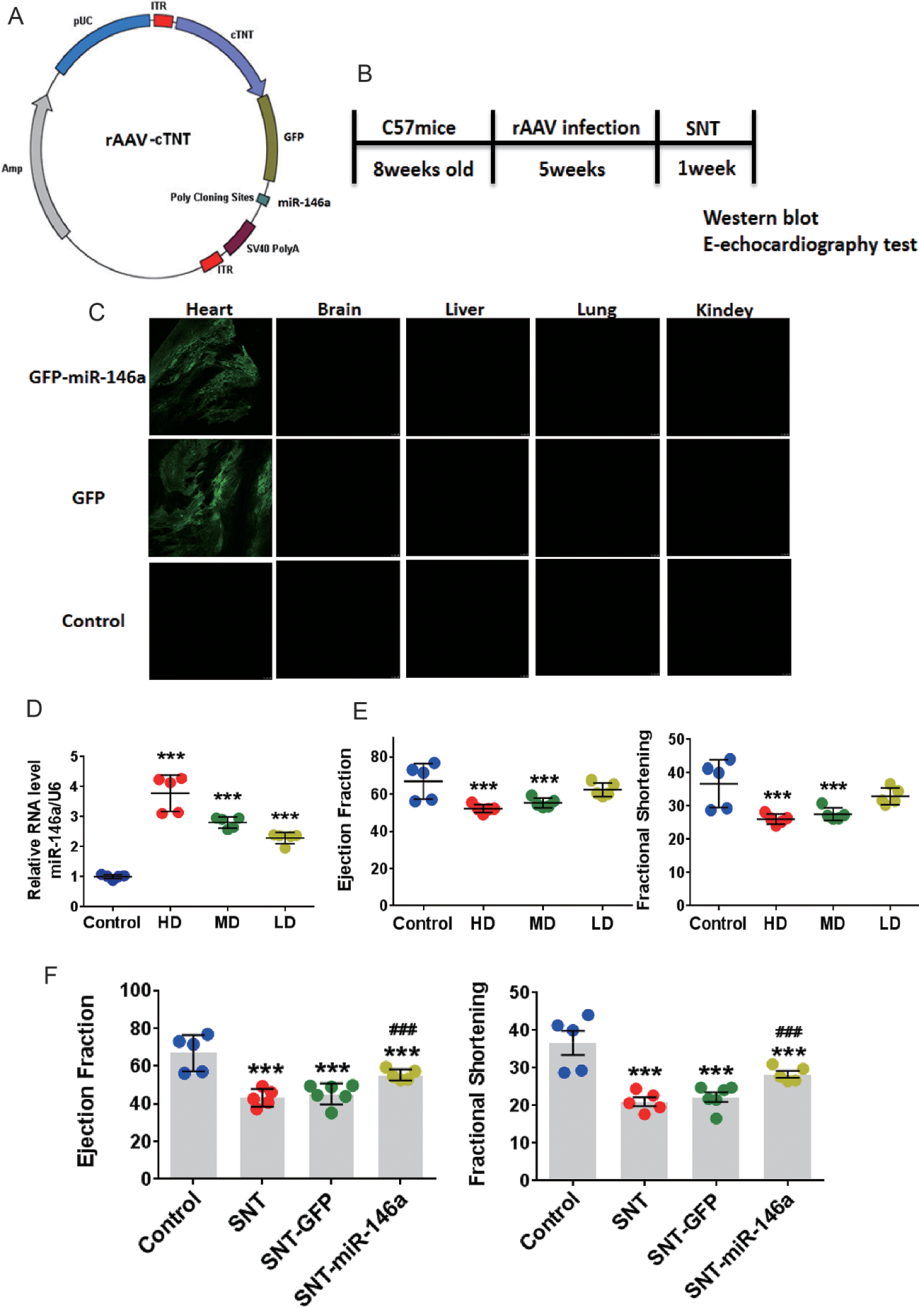
Overexpression of miR-146a attenuated cardiac contractile dysfunction induced by SNT *in vivo*. **(A)** Schematic view of AAV-9 designed to overexpress miR-146a. **(B)** Schedule for *in vivo* animal study. **(C)** Fluorescence intensity of GFP in different tissue sections captured by a fluorescence microscope (1×10^11^ viral particles). **(D)** MiR-146a levels after injection of different doses of AAV (HD, 10×10^11^; MD, 5×10^11^; LD, 1×10^11^ viral particles). **(E)** Echocardiography detection after injection of different dose virus. **(F)** Echocardiography detection after different treatments. ***P < 0.001 *vs* Control; ^###^P < 0.001 *vs* SNT-GFP.

Cardiac-specific overexpression of miR-146a was further induced to investigate the role of miR-146a in SNT-induced cardiotoxicity. Mice were treated with AAV9 carrying miR-146a, cardiac-specific cTNT promoters, and GFP (**Figure 3A**). Fluorescence images of different tissues indicated that the heart was exclusively transfected with virus after *i.v.* injection of the viral vectors (**Figure 3C**). To determine the optimal dose of AAV9, we detected the expression of miR-146a in the heart after *i.v.* injection of high (10×10^11^ viral particles), middle (5×10^11^ viral particles), and low (1×10^11^ viral particles) doses of AAV9 vectors. RT-PCR analysis revealed a dose-dependent overexpression of miR-146a in the heart (**Figure 3D**). Meanwhile, M-echocardiography results showed that overexpression of miR-146a after injection of high and middle doses of AAV9 vectors impaired cardiac contractile function (**Figure 3E**). Therefore, we chose 1×10^11^ viral particles as the optimal dose to treat mice, according to the schedule shown in **Figure 3B**. The cardiac contractile function was determined by echocardiographic examination after AAV9-mediated *in vivo* delivery of miR-146a. The results indicated that the presence of miR-146a in the heart markedly inhibited SNT-induced decreases in EF and FS, indicating improved cardiac function. The GFP vector (negative control) had no effect on SNT-induced changes in parameters of the cardiac contractile function (**Figure 3F**).

**Figure 4:**
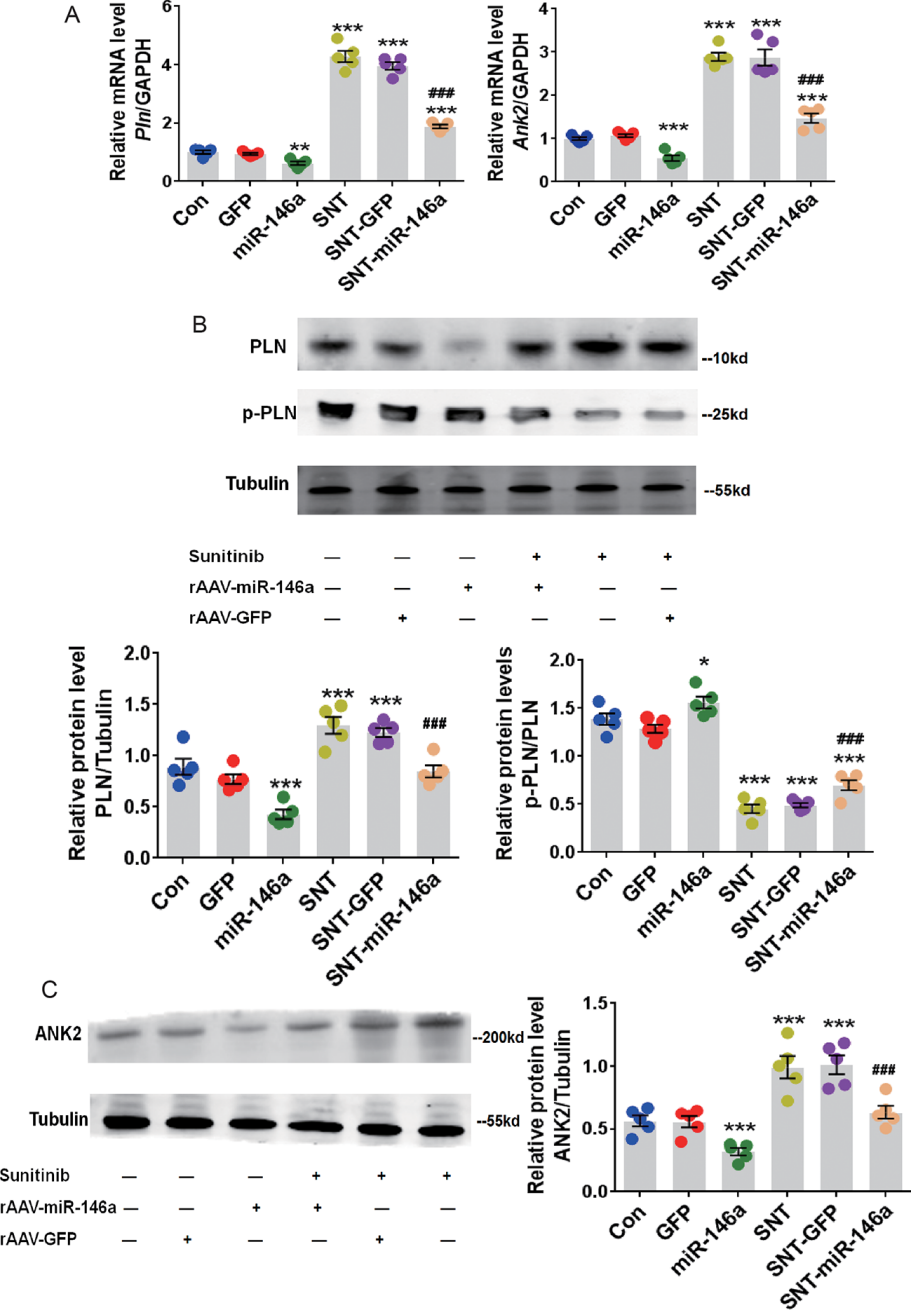
Upregulation of miR-146a decreased the abnormal expression of cardiac PLN and ANK2 caused by SNT. **(A)** *Pln* and *Ank2* mRNA levels in myocardium of mice with different treatments. Values are normalized to the level of GAPDH. **(B)** Representative Western Blot bands for PLN and p-PLN protein expression and corresponding summary data. Band densitometry was normalized to Tubulin. **(C)** ANK2 protein level was determined by western blot. Band densitometry was normalized to Tubulin. *P < 0.05, **P < 0.05,***P < 0.001 *vs* Control; ^###^P < 0.001 *vs* SNT-GFP.

The expression of PLN and ANK2 at the mRNA and protein levels were examined to determine whether these potential molecular targets mediate the cardioprotective effect of miR-146a. RT-qPCR data showed an apparent recovery of the mRNA expression of these two targets after overexpression of miR-146a and SNT administration (**Figure 4A**). In addition, SNT-induced elevated levels of PLN and ANK2 proteins were restored to normal levels after AAV9-miR-146a injection (**Figure 4B,C**). The level of p-PLN is of interest because through reversible phosphorylation, PLN can regulate SERCA. Because phosphorylation of PLN enhances the activity of SERCA, we determined the ratio of p-PLN/PLN and found that overexpression of miR-146a increased p-PLN/PLN in the treated group, compared to that in the control group. An increase in p-PLN/PLN indicated that the activity of SERCA was better protected (**Figure 4B**). In addition, we noticed that overexpression of miR-146a resulted in a significant decrease in PLN and ANK2 expression at the mRNA and protein levels (**Figure 4A,C**). To verify the specificity of the effect of miR-146a overexpression on calcium handling proteins, we determined the alterations of SERCA and RYR2 expression, which is a major Ca^2+^ release channel on the sarcoplasmic reticulum. The expression levels of SERCA and RYR2 proteins had no significant change 7 days after SNT administration (*P* > 0.05) (**Figure S2**). The result is consistent with our recent finding (Li et al., 2019). In addition, overexpression of miR-146a did not alter the expression levels of SERCA and RYR2 proteins (**Figure S2**). The result revealed a specific regulation of miR-146a on PLN and ANK2 expression.

### Overexpression of miR-146a Alleviates SNT-Induced Contractile Dysfunction *In Vitro*

**Figure 5:**
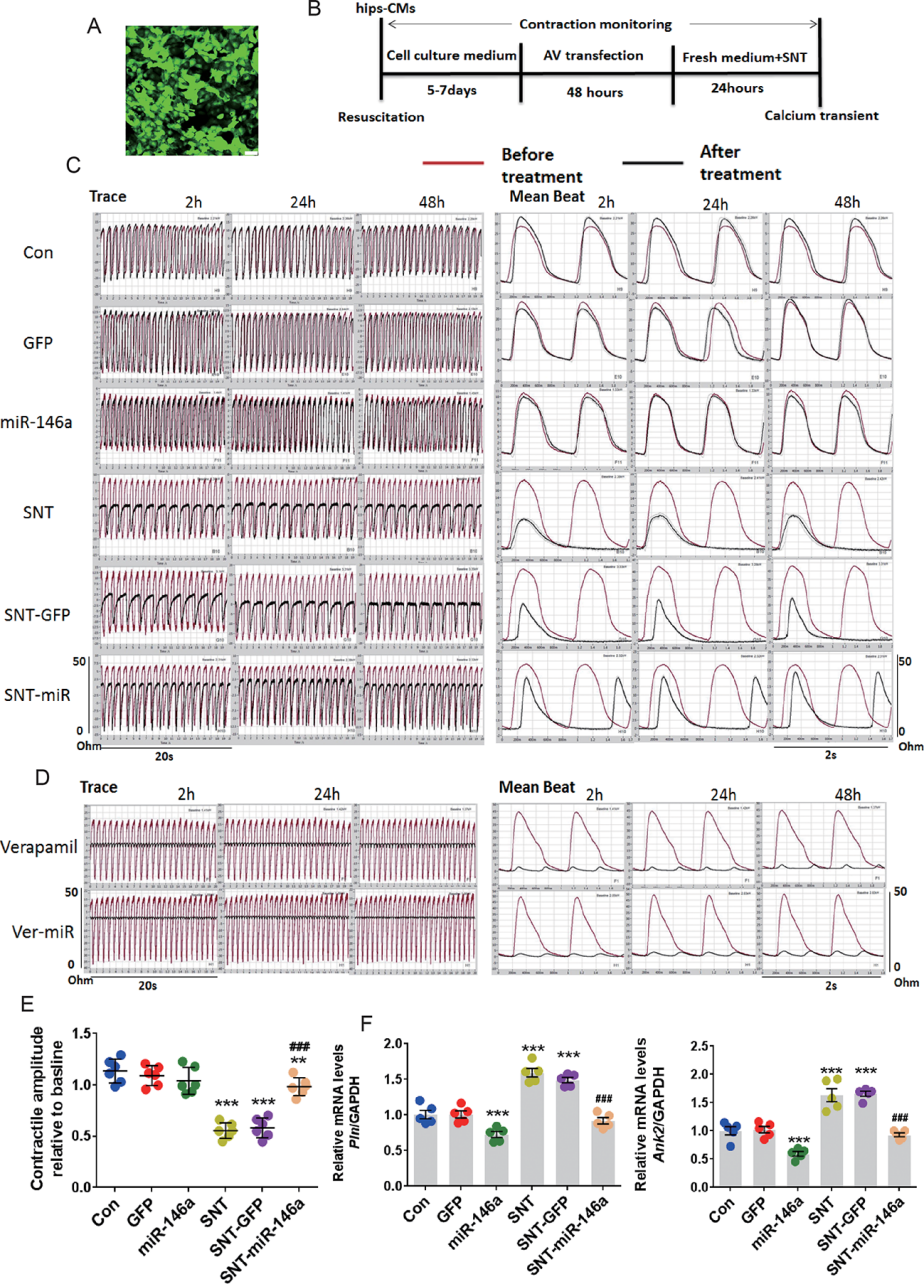
MiR-146 overexpression alleviated SNT-induced contractile dysfunction in hiPSC-CMs. **(A)** Human induced pluripotent stem cell-derived cardiomyocytes (hiPSC-CMs) was successfully infected with AV when the MOI was 10. **(B)** The schedule for hiPSC-CMs treatment. **(C)** Representative contractile traces and mean beat signals of hiPSC-CMs with different treatment (SNT, 3 μmol). **(D)** Representative contractile traces and mean beat signals of hiPSC-CMs after exposure to verapamil (100 nM). **(E)** Summary data for contractile amplitude of hiPSC-CMs relative to baseline. **(F)** *Pln* and *Ank2* mRNA levels based on the different treatments. Values are normalized to the level of GAPDH. **P < 0.01, ***P < 0.001 *vs* Control; ^###^P < 0.001 *vs* SNT-GFP.

The cardioprotective effect of miR-146a was further confirmed by measuring cellular contractility in hiPSC-CMs using CardioExcyte 96 (see *Materials and Methods*). The validity of the measurement was confirmed by a positive control, doxorubicin, and a negative control, axitinib, which have been shown to exert no cardiac side effect (Sharma et al., 2017) (**Figure S3**). A recombinant AV vector was used to transfect hiPSC-CMs at several MOI values (5, 10, 30, 50, and 100). At MOI 10, nearly 100% of cells were successfully transfected (**Figure 5A**). We plated hiPSC-CMs in a CardioExcyte 96 Sensor plate (3×10^4^ cells/well), transfected the cells with virus vector, added SNT 48 h after transfection, and checked the contractile amplitude of hiPSC-CMs (**Figure 5B**). Compared to that in the control, overexpression of AV-miR-146a and AV-GFP did not change the contractile amplitudes. However, SNT caused a significant decrease in contractile traces within 2 h. The corresponding mean beat data are shown in **Figure. 5C**. The result clearly showed that overexpression of miR-146a significantly alleviated SNT-induced decreases in contractile amplitude of hiPSC-CMs (**Figure 5E**). Moreover, abnormal mRNA levels of *Pln* and *Ank2* returned to normal levels after miR-146a overexpression (**Figure 5F**). Next, the specificity of the effect of miR-146a overexpression on contractile suppression was examined. Verapamil is an L-type calcium channel inhibitor; at 100 nM, it evoked a similar inhibitory effect on contractile amplitude as that of SNT (**Figure 5D**). Overexpression of miR-146a did not antagonize the decline in contractility caused by verapamil (**Figure 5D**). These results indicated that overexpression of miR-146a specifically alleviated SNT-induced contractile dysfunction in hiPSC-CMs.

**Figure 6:**
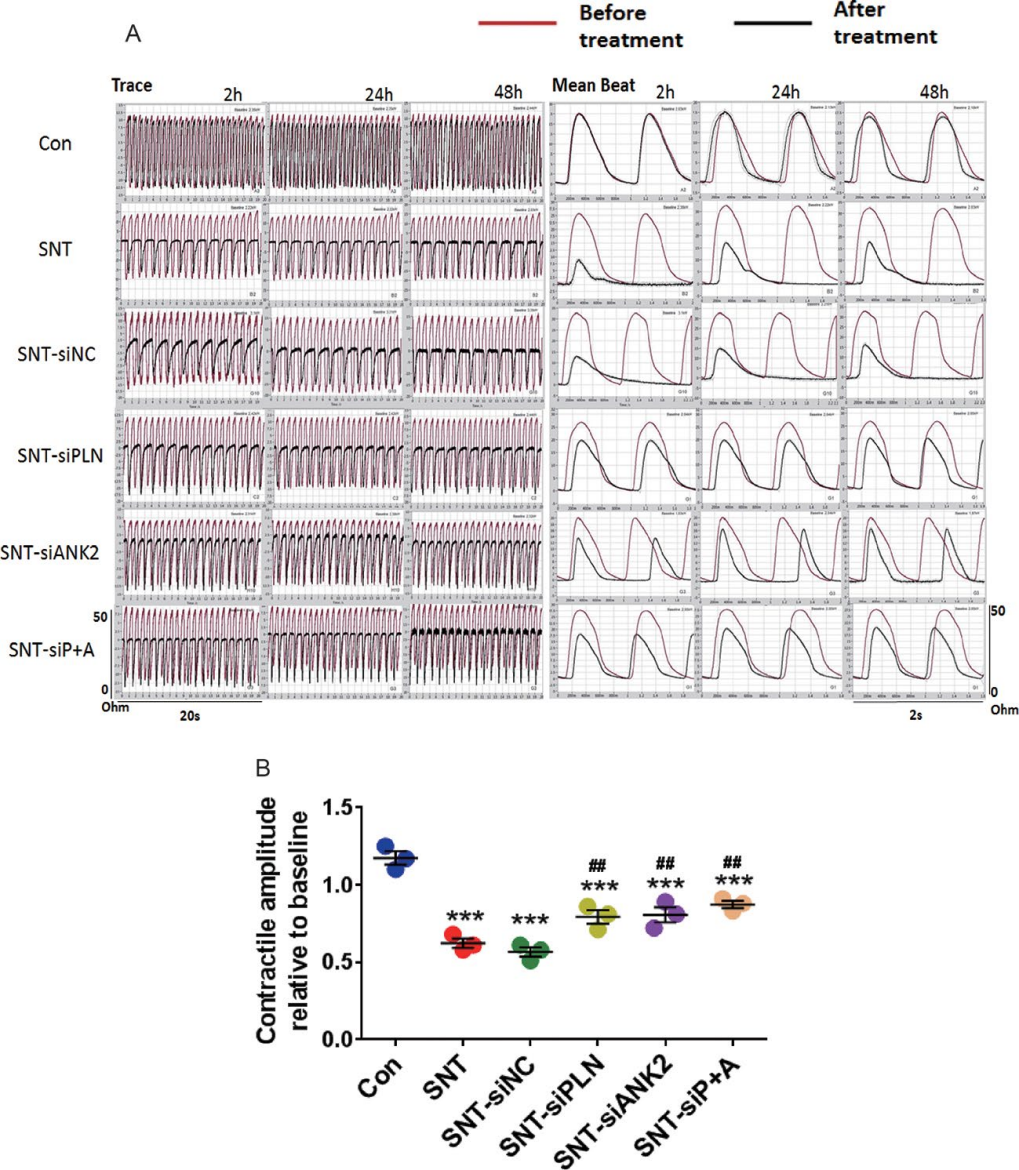
Knockdown of either PLN or ANK2 alleviated SNT-induced contractile suppression in hiPSC-CMs. **(A)** Representative contractile traces and mean beat signals of hiPSC-CMs. **(B)** Summary data for contractile amplitude under different treatments. ***P < 0.001 *vs* Control; ^##^P < 0.01, *vs* SNT-siNC.

Next, to further verify that miR-146a evoked its effect by targeting PLN and ANK2, we observed the effect of PLN or ANK2 knockdown on cardiac contractility in hiPSC-CMs using small interference RNA (siRNA). First, the efficacy of the knockdown was tested by RT-PCR using three different siRNA sequences, two of which effectively downregulated the mRNA expression of PLN or ANK2, whereas the scramble siRNA (SiNC) had no effect on PLN or ANK2 mRNA expression (**Figure S4**). The siRNA sequence with the most efficient knockdown was used for the subsequent experiment. Next, we assessed the effect of PLN or ANK2 knockdown on the contractility of hiPSC-CMs. As shown in **Figure 6**, knockdown of either PLN or ANK2 did not affect the contractile amplitudes, compared to that in the control cells. Knockdown of either PLN or ANK2 significantly alleviated SNT-induced contractile suppression. However, knockdown of both PLN and ANK2 did not provide additive cardioprotective effect. The result provided further evidence supporting our notion that miR-146a mediates its effect *via* PLN or ANK2 regulation.

**Figure 7:**
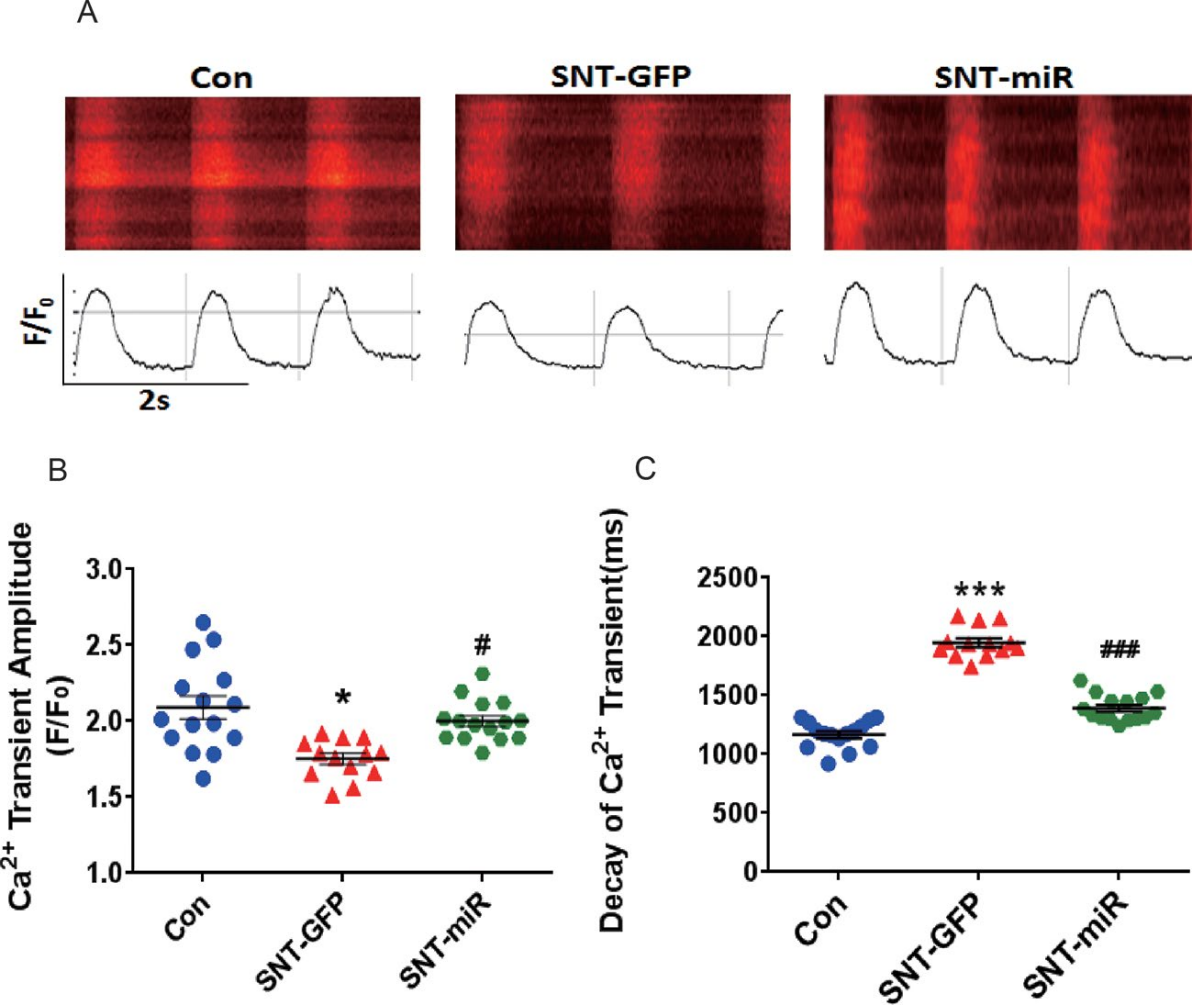
MiR-146 overexpression alleviated SNT-induced calcium transient anomaly in hiPSC-CMs. **(A)** Typical image of calcium transient. **(B)** Summary data for Ca^2+^ transient amplitude(F/F_0_). **(C)** Decay of Ca^2+^ transient time(ms). *P < 0.05, *** P < 0.001 *vs* Control, ^#^P < 0.05, ^###^ P < 0.001 *vs* SNT-GFP group.

Next, we observed calcium transients in hiPSC-CMs. Spontaneous calcium transients in hiPSC-CMs were recorded with a laser-scanning confocal microscope in line-scan mode (**Figure 7A**). Compare to the control, SNT (3 µM) remarkably reduced calcium transient amplitude and prolonged the decay of calcium transients, whereas overexpression of miR-146a prevented SNT-induced effect on calcium transients (**Figure 7B, C**). Our data indicated that overexpression of miR-146a prevented the deteriorating effect of SNT on calcium handling in hiPSC-CMs.

## Discussion

To date, information on alterations in the expression of miRNAs following SNT treatment is limited. Some reports have provided evidence that miR-221, miR-222, and miR-101 may be promising targets of SNT in renal cell carcinoma (Khella et al., 2015; Goto et al., 2016). Rainer et al. (2012) have performed a comprehensive analysis of alterations in the expression of microRNAs in cultured HL-1 cells, a murine cardiomyocytic cell line; however, they did not find any differential expression of microRNA after exposure of cells to SNT (approximately 1.88 μmol) for 24 h (Rainer et al., 2012). Contrary to the previous observation, our study revealed that SNT evoked a significant downregulation of miR-146a in the myocardium of mice after exposure to SNT when cardiac contractile dysfunction was evident. The decreased expression of miR-146a was further validated by quantitative RT-qPCR. The different response/sensitivity to the drug between cultured cell line and native myocardium may contribute to the discrepancy between these results.

MiR-146 is a family of miRNA precursors including miR-146a and miR-146b in mammals, which are transcribed from different genes on chromosomes 5 and 10. MiR-146a has been primarily identified as an immune system regulator (Ordas et al., 2013). A previous study has shown that miR-146a attenuates sepsis-induced cardiac dysfunction (Gao et al., 2015). Our *in vivo* and *in vitro* results indicated that upregulation of miR-146a exerted cardioprotective effect against SNT-induced contractile dysfunction. However, a recent study found that miR-146a was upregulated in failing cardiomyocytes and that overexpression of miR-146a suppresses the expression level of small ubiquitin-like modifier 1 (SUMO1), which reduces SERCA2a SUMOlation and finally leads to cardiac contractile dysfunction (Oh et al., 2018). The findings suggest that there are distinct alteration patterns of miR-146a in different pathological conditions. Alteration in miRNA expression can influence the expression of its target gene(s). In the current study, we focused on two potential targets of miR-146a, *Pln* and *Ank2*, which have been shown to participate in calcium cycle homeostasis (Zvaritch et al., 2000; Mohler et al., 2005). Luciferase assay results showed that miR-146a directly regulated *Pln* and *Ank2* and led to overexpression of miR-146a through AAV9-mediated gene delivery, resulting in a significant decrease in the mRNA and protein expression of PLN and ANK2 in mouse myocardium. Upregulation of PLN and ANK2 at the mRNA and protein levels was observed in the myocardium of mice treated with SNT. Considering these results, we concluded that SNT upregulated PLN and ANK2 through miR-146a downregulation.

Cardiac contractility depends on fine-tuned excitation–contraction coupling. During each contractile cycle, action potential activates voltage-dependent L-type calcium channel, which in turn triggers sarcoplasmic reticulum (SR) Ca^2+^ release through RYR2. The combination of Ca^2+^ influx through LTCC and Ca^2+^ release from RYR2, known as calcium transient, rises intracellular Ca^2+^ concentration ([Ca^2+^]_i_) and activates the contractile machinery. For relaxation to occur, Ca^2+^ is mainly removed from the cytosol by SERCA pump with minor contribution from sarcolemmal Na^+^/Ca^2+^ exchange. PLN is well known as a prominent regulator of myocardial contractility through regulation of SERCA activity. Dephosphorylated PLN is an inhibitor of SERCA, and phosphorylation of PLN relieves this inhibition. An early study has already shown that PLN-overexpressing mice are associated with inhibition of Ca^2+^ transient and left ventricular contractile function (Kranias and Hajjar, 2012). On the contrary, partial or complete ablation of PLN in mouse models is associated with increases in SR Ca^2+^ transport and enhanced myocardial contractility (Luo W, 1994; Luo et al., 1996). On the other hand, the ANK2 is an adaptor protein primarily localized at the myocyte M-line and transverse-tubule membranes, where it associates with select membrane and signaling proteins to regulate excitation–contraction coupling (Koenig and Mohler, 2017). Partial ablation of ANK2 in mouse models is associated with increases in Ca^2+^ transients and SR Ca^2+^ content (Camors et al., 2012). In our study, SNT evoked a significant suppression on Ca^2+^ transients and cellular contractility in hiPSC-CMs, and overexpression of miR-146a prevented the SNT-induced reduction in calcium transients. In addition, cardiac-specific overexpression of miR-146a protected the heart from a decrease in LVEF in SNT-treated mice, along with an evident recovery in the expression of PLN and ANK2. Moreover, specific knockdown of PLN or ANK2 using small interference RNA produced a similar cardioprotective effect as that produced by miR-146a overexpression in hiPSC-CMs. Overexpression of miR-146a did not alter the expression levels of SERCA and RYR2 proteins. These findings supported the notion that overexpression of miR-146a protects the heart against SNT-induced contractile dysfunction by targeting PLN and ANK2. However, the effect of deterioration on the cardiac contraction at high doses of miR-146a was observed in the study (**Figure 3E**) and it may result from excessive expression of miR-146a, which may change other genes expression and thus lead to unexpected effects. The result suggests that the dosage of this therapy needs to be tightly controlled. Moreover, knockdown of both PLN and ANK2 did not result in additive cardioprotective effect in hiPSC-CMs. The data indicated that the two target proteins of miR-146a may eventually contribute to the same signaling pathway of intracellular calcium hemostasis.

There were some limitations in the present study. First, we only assessed the role of miR-146a, which was one of differential expression miRNAs (see **Figure 1C**). The partial recovery of cardiac contractility through overexpression miR-146a suggested that other miRNAs or other mechanism may be involved in SNT-induced cardiac dysfunction. Second, we did not evaluate the effect of miR-146a on cardiac dysfunction induced by other tyrosine kinase inhibitors. Presently, the universality of the cardioprotective effect of miR-146a for tyrosine kinase inhibitors is unknown.

In summary, the results of our *in vivo* and *in vitro* experiments showed that sunitinib downregulated miR-146a, which contributes to cardiac contractile dysfunction by targeting PLN and ANK2, and that upregulation of miR-146a alleviated the inhibitory effect of SNT on cardiac contractility. Thus, miR-146a could be a useful agent with protective effect against sunitinib-induced cardiac dysfunction.

## Ethics Statement

This study was carried out in accordance with the recommendations of Hebei Medical University Ethics Committee.

## Author Contributions

LS, CL, HZ, SQ, and TF performed the research. YX designed the study. LS and CL analyzed the data. LS and YX wrote the manuscript.

## Funding

This work was supported by the National Natural Science Foundation of China (31771259 to Y.X.) and the Science and Technology Foundation of Hebei Province (17274803D to Y.X.).

## Conflict of Interest Statement

The authors declare that the research was conducted in the absence of any commercial or financial relationships that could be construed as a potential conflict of interest.
