# Supplementary material for: Downregulation of miR-146a Contributes to Cardiac Dysfunction Induced by the Tyrosine Kinase Inhibitor Sunitinib
Source: Front Pharmacol. 2019 Aug 23;10:914. doi: 10.3389/fphar.2019.00914 (PMC6716347; doi:10.3389/fphar.2019.00914)
Supplement: Supplementary file 1 [file DataSheet_1.pdf]

Supplementary Materials

Downregulation of miR-146a Contributes to Cardiac Dysfunction Induced by the Tyrosine Kinase Inhibitor Sunitinib

Li Shen[1], Congxin Li[1], Hua Zhang[1], Suhua Qiu[1], Tian Fu and Yanfang Xu[1]*

[1]Department of Pharmacology, Hebei Medical University; The Key Laboratory of New Drug Pharmacology and Toxicology, Hebei Province; The Key Laboratory of Neural and Vascular Biology, Ministry of Education, Shijiazhuang 050017, China

*** Correspondence:**

Yanfang Xu

yanfangxu@hebmu.edu.cn

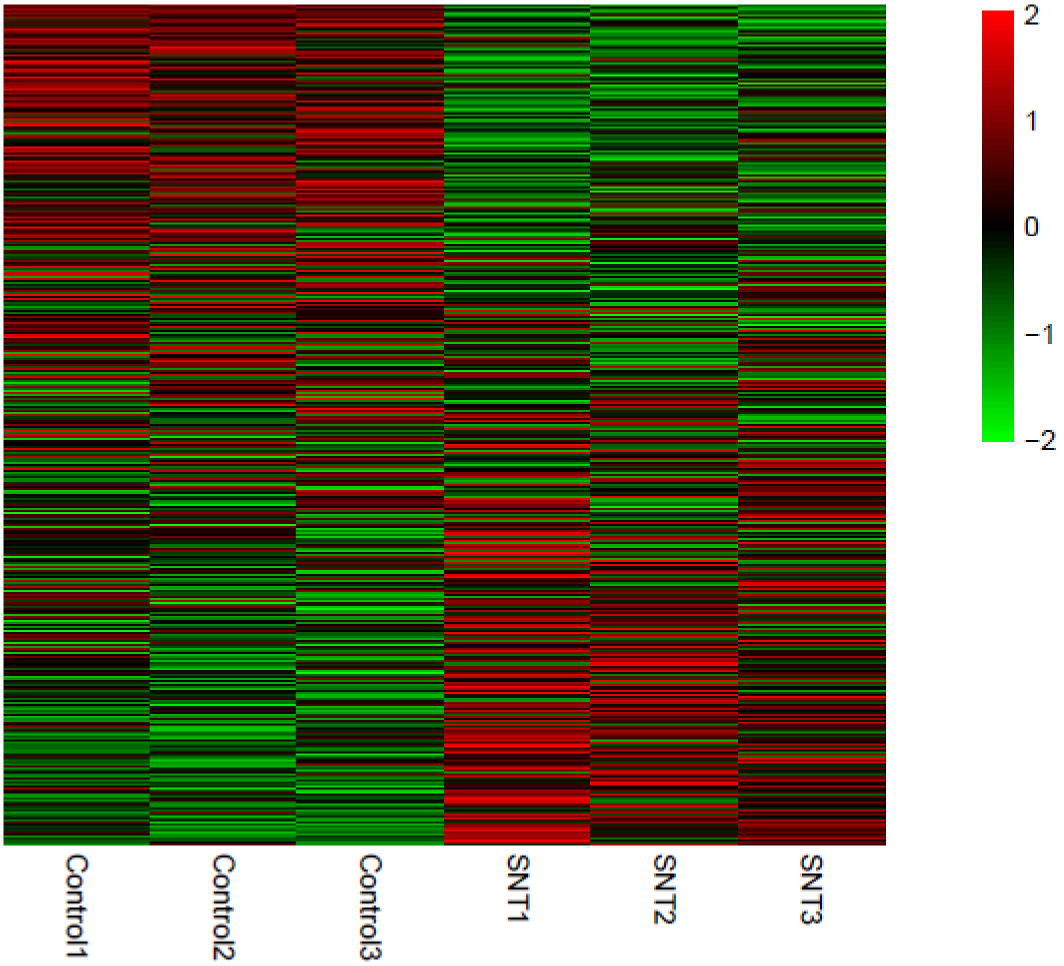

Fig.S1. Complete microarray heatmap of miRNAs. Microarray heatmap illustrating differentially expressed miRNAs in the left ventricular of mice after treatment of SNT 40 mg/kg for 1 week. Red indicates high gene expression, and blue indicates low gene expression. The datasets of microarray can be found in the GEO repository (GSE125952). The GEO website link is https://www.ncbi.nlm.nih.gov/geo/query/acc.cgi?acc.

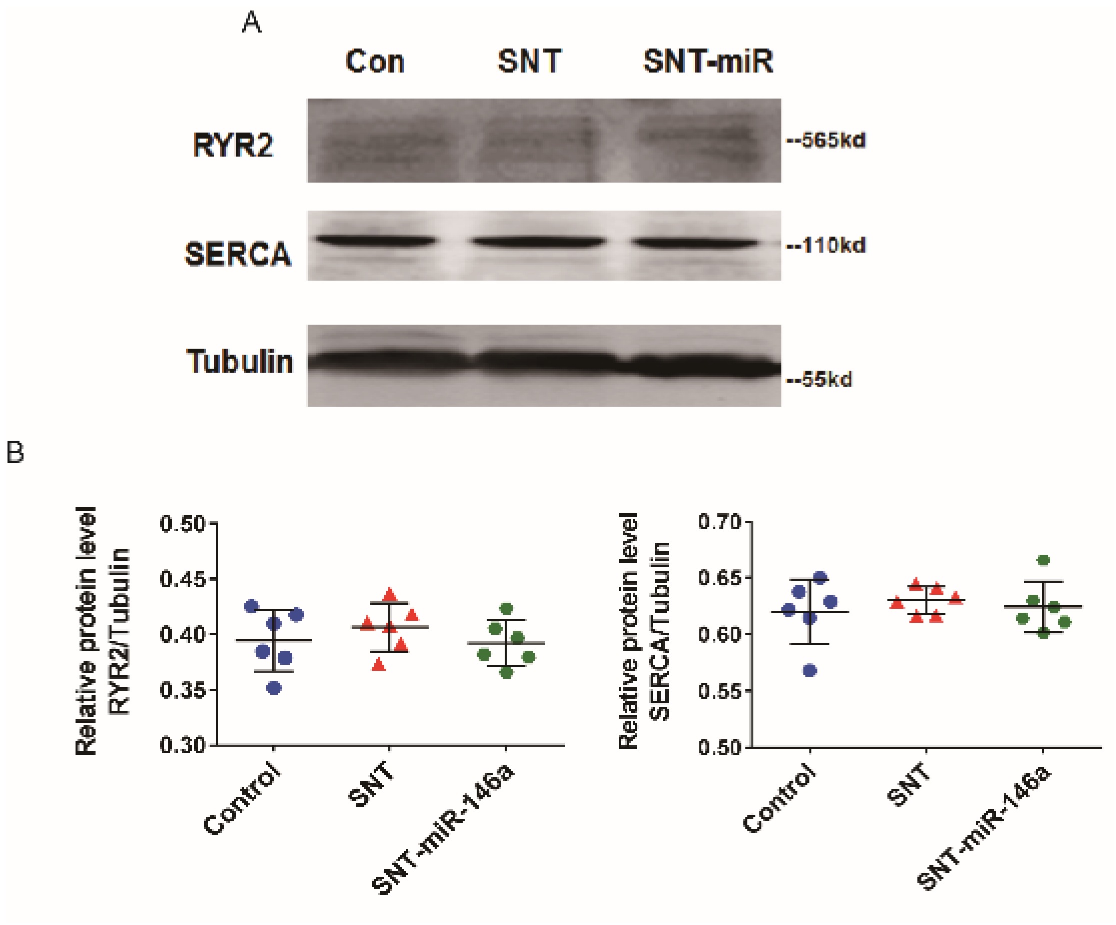

Fig.S2. Effect of miR-146a overexpression on calcium handling proteins. (A) Representative Western Blot bands for RYR2 and SERCA protein expression; (B) Corresponding summary data. Band densitometry was normalized to Tubulin.

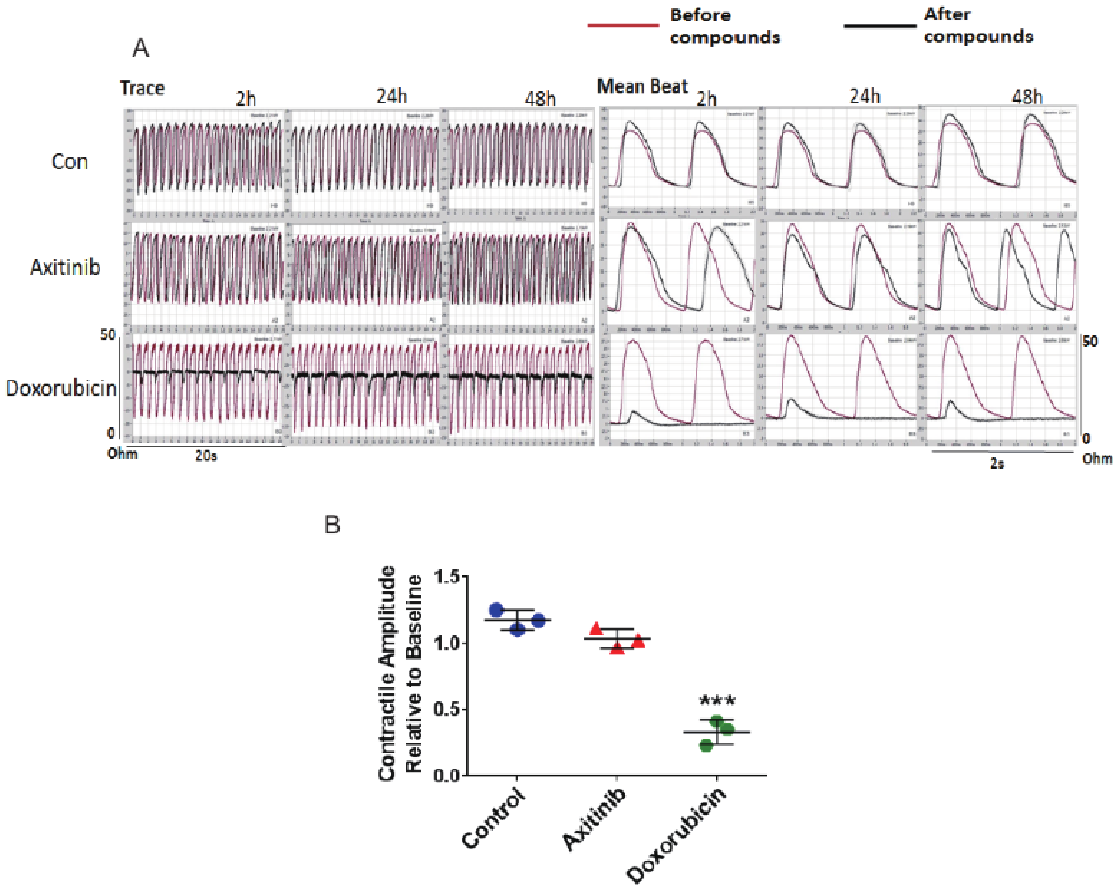

Fig. S3. Validation of the measurement for the contraction of hiPSC-CMs. (A) Representative contractile traces and mean beat signals of hiPSC-CMs after exposure to a positive control, doxorubicin (1 μM) and a negative control, axitinib (1 μM). (B) Summary data for **c**ontractile amplitude of hiPSC-CMs relative to baseline. $^{***}P < 0.001$ *vs* Control.

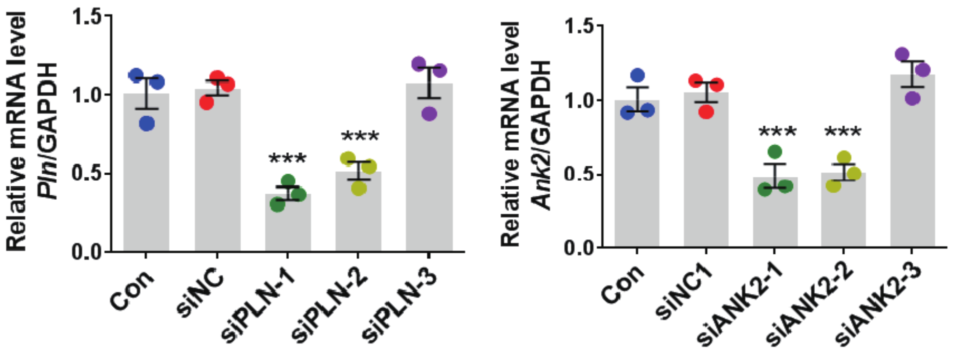

Fig.S4. Effect of siRNA knockdown on the mRNA expression of of PLN or ANK2.

SiPLN (or siANK2)-1,2,3 represented three different siRNA sequences, respectively.

$^{***}P < 0.001$ *vs* Control.
